# Interdisciplinary biophysical studies of membrane proteins bacteriorhodopsin and rhodopsin

**DOI:** 10.1007/s12551-022-01003-y

**Published:** 2022-10-08

**Authors:** Karim Fahmy, Thomas P. Sakmar

**Affiliations:** 1 Institute of Resource Ecology, Biophysics Division, Helmholtz-Zentrum Dresden-Rossendorf e.V. (HZDR), 01328 Dresden, Germany; 2 Laboratory of Chemical Biology and Signal Transduction, Rockefeller University, New York, NY 10065 USA

**Keywords:** Bacteriorhodopsin, Rhodopsin, Signal transduction, G protein–coupled receptor, Spectroscopy

## Abstract

The centenary of the birth of H. Gobind Khorana provides an auspicious opportunity to review the origins and evolution of parallel advances in biophysical methodology and molecular genetics technology used to study membrane proteins. Interdisciplinary work in the Khorana laboratory in the late 1970s and for the next three decades led to productive collaborations and fostered three subsequent scientific generations whose biophysical work on membrane proteins has led to detailed elucidation of the molecular mechanisms of energy transduction by the light-driven proton pump bacteriorhodopsin (bR) and signal transduction by the G protein–coupled receptor (GPCR) rhodopsin. This review will highlight the origins and advances of biophysical studies of membrane proteins made possible by the application of molecular genetics approaches to engineer site-specific alterations of membrane protein structures.

## Introduction and historical notes

During this centenary year of the birth of the great molecular and chemical biologist, H. Gobind Khorana (1922–2011), much will be written about his contributions to nucleotide synthesis methodology and the elucidation of the genetic code, for which he shared the Nobel Prize in Physiology or Medicine in 1968. He is also recognized as a pioneer of “synthetic biology” because of his report of the first chemical synthesis of a functional gene (Khorana 1979). However, during the early 1970s, the idea of “starting anew” in an entirely new field began “to take hold” in Khorana. As he himself wrote, “I began to think about biological membranes with the distant hope that I might get into areas of molecular neurobiology and signal transduction” (Khorana). In a letter written at M.I.T. to F. H. C. Crick dated May 22, 1974, Khorana states, “I have…become very deeply interested in the chemistry of membranes and have had a small group working in this field.” He goes on to state, “I have definitely concluded that this sort of work is a reasonable starting point…” (Khorana 1974) (letter from HGK to Crick).

More than a decade earlier in 1961, Peter Mitchell proposed the hypothesis that a proton gradient across cell membranes was central to energy transduction—the so-called chemiosmotic hypothesis (Mitchell 1961). But at the time, it was not clear how such a proton gradient could be established or maintained. Starting in about 1971, W. Stoeckenius made a series of discoveries that would provide the basis for an attractive and relatively straightforward system to study proton translocation from the inside to the outside of a cell, a central requirement of the Mitchell hypothesis. D. Oesterhelt and Stoeckenius found that the “purple membrane” of the halophilic archaebacterium *H. halobium* was made up of a single membrane protein, an opsin-like protein with a retinylidene chromophore called bacteriorhodopsin (bR) (Oesterhelt and Stoeckenius 1971). Under relatively anaerobic conditions, bR forms patches of two-dimensional crystalline arrays in the cell membrane and serves as a light-driven proton pump to create an electromotive force that can be coupled to ATP synthesis. In a classic experiment in membrane biochemistry, which energized the field, E. Racker and Stoeckenius reconstituted a membrane vesicle system containing bR with ATPase and showed light-dependent ATP synthesis (Racker and Stoeckenius 1974).

**Fig. 1:**
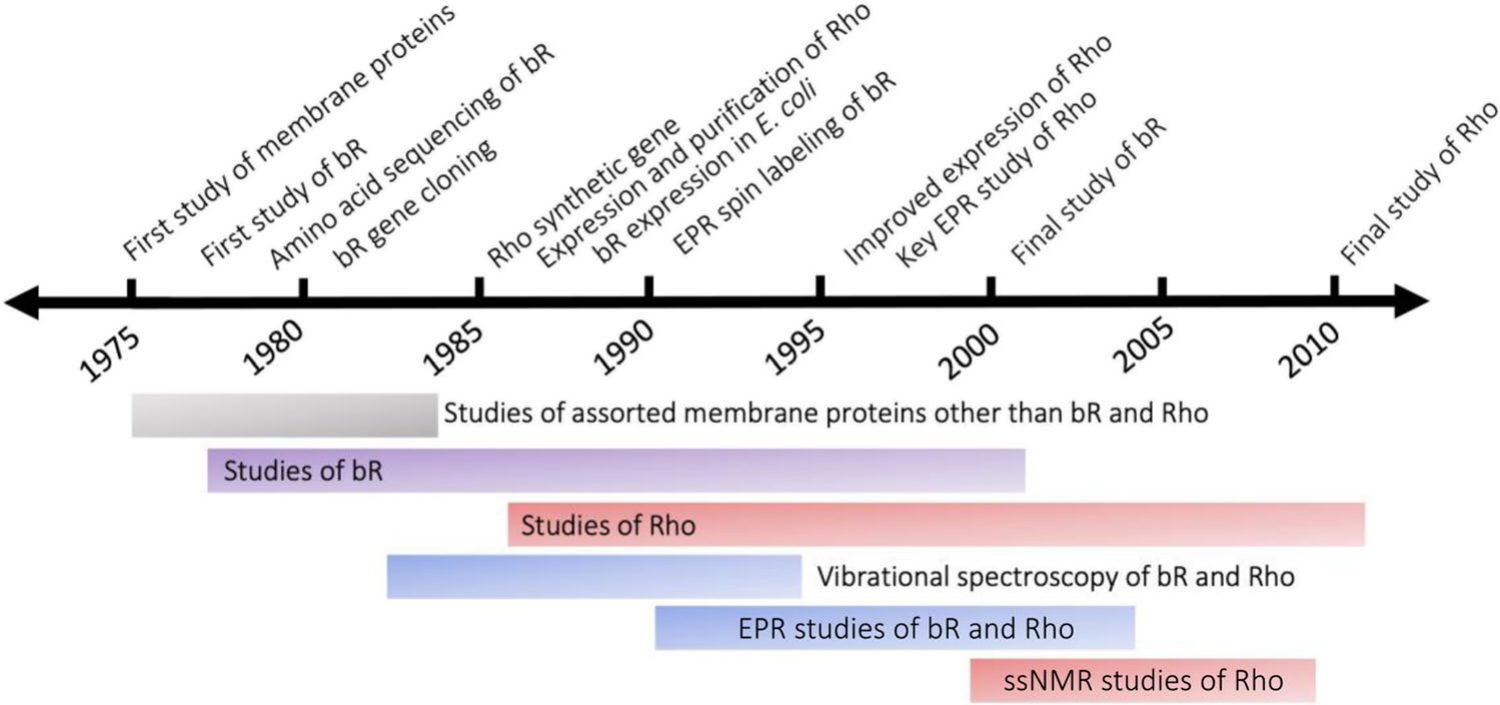
Career timeline showing the period from 1975 to 2011 when H. G. Khorana published scientific papers on membranes and membrane proteins. Selected events are shown at the top. The span of publications on bR, rhodopsin, and assorted other membrane proteins is shown below along with the span of papers in three key areas of biophysical methods: vibrational spectroscopy, EPR spectroscopy, and solid state NMR spectroscopy

Studies in the late 1970s established the foundations of the bR photocycle (Stoeckenius and Bogomolni 1982), while R. Henderson and P. N. T. Unwin pioneered electron diffraction methods to create projection maps of the two-dimensional crystals of bR found in purple membrane that suggested the presence of seven protein densities traversing the membrane (Henderson and Unwin 1975). It was at about this time, with encouragement and help from both Racker and Stoeckenius, that Khorana decided to put aside studies on other membrane proteins such as glycophorin A and cytochrome b5 and focus his attention clearly on bR. About a decade later, a broadening of Khorana’s focus led to studies of the visual pigment rhodopsin, another light-absorbing membrane protein with a retinal (vitamin A aldehyde) chromophore. In the sections below, we will discuss the application of interdisciplinary biophysical methods that Khorana and his colleagues and collaborators used to study bR and rhodopsin (Table 1). The review will cover the period from the mid-1970s until about 2010 when molecular biological methods were used in tandem with biophysical techniques to advance structure–activity studies, but before high-resolution X-ray and cryo-electron microscopy (EM) methods were widely used for membrane protein structure determination (Fig. 1). The primary aim of the review is to place the pioneering interdisciplinary biophysical contributions of the Khorana laboratory into the proper perspective within the nascent field of quantitative studies of membrane proteins.

**Table 1:** Selected biophysical methods used to study recombinant bR and rhodopsin mutants

Mass spectrometry
Time-resolved laser flash photolysis absorption spectroscopy
Linear dichroism measurements
Time-resolved photovoltage measurements
Resonance Raman spectroscopy
Microprobe resonance Raman spectroscopy
Fourier-transform IR (FTIR) spectroscopy
Time-resolved FTIR spectroscopy
Electron paramagnetic resonance (EPR) spectroscopy
Time-resolved site-directed EPR spectroscopy
Double electron–electron resonance (DEER) EPR spectroscopy
^31^P-nuclear magnetic resonance (NMR) spectroscopy
^19^F-NMR spectroscopy
Magic-angle spinning solid state NMR spectroscopy

## Studies of the light-driven proton pump bacteriorhodopsin (bR)

**Fig. 2:**
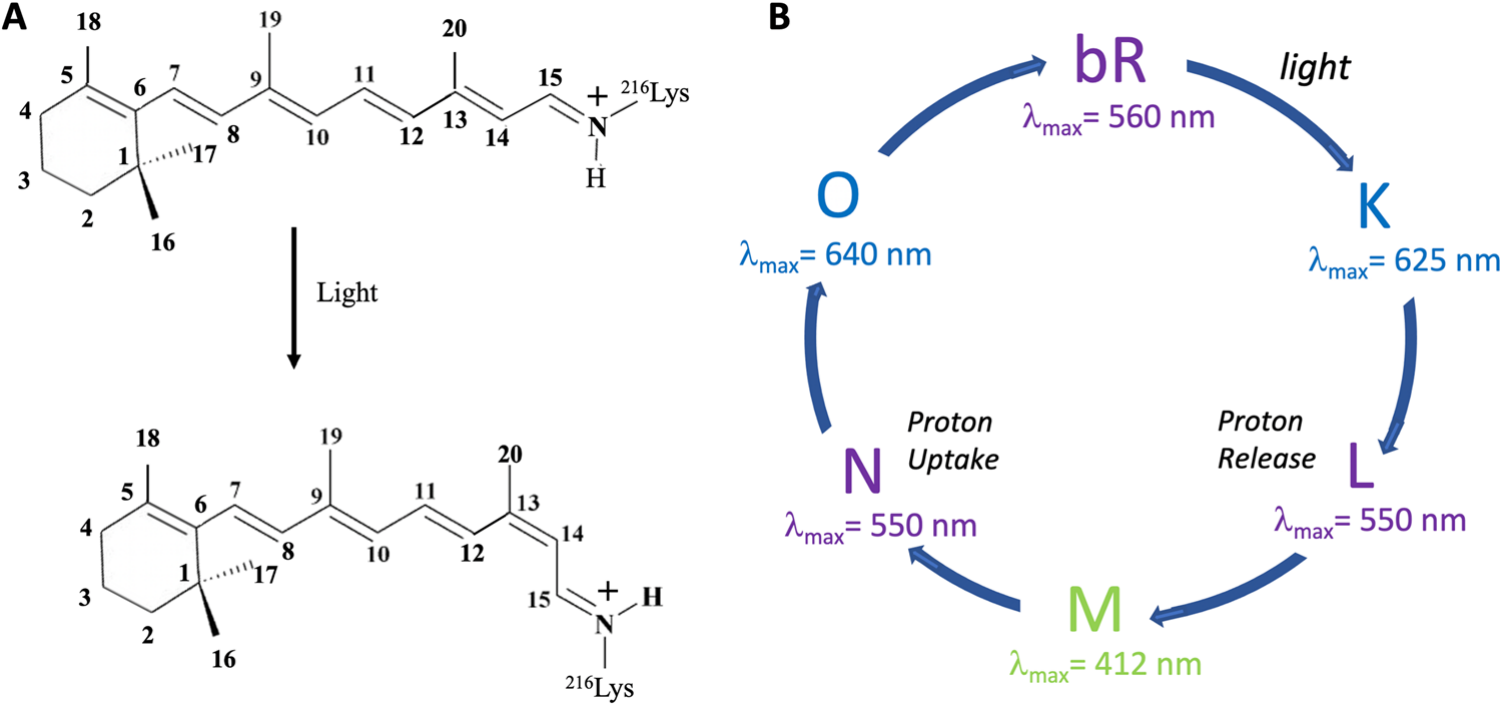
Principles of bR function. **A** The chromophore in bR is all-*trans*-retinal, which photoisomerizes to 13-*cis*-retinal. **B** The key intermediates in the photocycle of bR are presented along with their wavelengths of maximal absorption

As noted above, bR is a primitive single-molecule light-powered system that generates a transmembrane proton gradient. With the eluciation of the bR photocycle, the next problem was to identify specific amino acid residues that participated in proton translocation and those that played a role in the deprotonation of the retinylidene-opsin Schiff bases linkage in the first half of the cycle, and its reprotonation in the second half of the cycle (Fig. 2). However, the primary structure of bR was not known, and because of the extreme hydrophobic nature of integral membrane proteins, the techniques established for water-soluble peptide sequencing were not readily applicable to the challenge. At the time that Khorana reported the primary structure determination of bR, not a single integral membrane protein had been sequenced. In collaboration with K. Biemann, methods that included mass spectrometry in combination to Edman degradation were developed to sequence hydrophobic peptides, and the complete amino acid sequence of bR was reported. Peptides derived from bR that were not amenable to Edman degradation, including many very hydrophobic peptides, were reduced to polyaminoalcohols to facilitate gas chromatography mass spectrometry analysis (Gerber et al. 1979). Soon thereafter, the gene for bR from *H. halobium* was cloned and sequenced, and remarkably, the deduced amino acid sequence of bR was identical to the sequence determined earlier using laborious peptide sequencing methods (Dunn et al. 1981; Gerber et al. 1979).

With the report and confirmation of the primary structure of bR, work could commence on determining the linkage site for the retinal chromophore, which would help define its absolute orientation in bR with respect to the membrane and provide a foundation for subsequent structure–activity studies. The chromophore was unambiguously determined to be coupled to lysine 216 (Bayley et al. 1981). Having the primary structure also allowed the predication of secondary structure models, which resulted in the proposal that bR comprises seven-transmembrane helical segments, which was consistent with the low-resolution project model from the earlier electron diffraction studies (Khorana et al. 1979). With the development for biochemical methods to isolate bR from purple membrane and then reconstitute it in vesicle for proton pumping assays in defined conditions, the stage was set for an extended series of structure–activity studies (Huang et al. 1980). Additional studies showed that bR could be completely denatured and then refolded in detergent phospholipid mixtures (Huang et al. 1981). Subsequently, to facilitate site-directed mutagenesis by restriction fragment replacement, which overcame many of the limitations of mismatch-oligonucleotide-directed site-specific mutagenesis, an artificial gene for bacterio-opsin was designed and synthesized for optimized heterologous expression in *E. coli* (Lo et al. 1984; Nassal et al. 1987).

The synthesis of the gene for bacterio-opsin from *Halobacterium halobium* and its expression in *E. coli* and subsequent reconstitution with the native chromophore all-trans retinal (Braiman et al. 1987; Karnik et al. 1990) facilitated the onset of biochemical and biophysical studies that led to fundamental discoveries of membrane protein structure–function relationships. In parallel, spectroscopic techniques were advanced, which quickly exploited the novel genetic engineering tools to resolve site-specific and atomistic details of the conformational transitions that underlie light-driven proton translocation. Among these techniques, Fourier-transform infrared (FTIR) spectroscopy probably experienced the largest leap in evolving from a routine quality control method for organic synthesis to an advanced biophysical technique for the label-free detection of membrane protein conformational changes on time scales from nanoseconds to minutes (Rodig et al. 1999; Siebert and Hildebrandt 2008). The chemical information provided by the determination of group-specific vibrational frequencies also motivated hypothesis-driven mutagenesis studies with an entirely new paradigm: genetic engineering aimed at producing a spectroscopic phenotype rather than a physiologic phenotype.

It was this synergistic interplay between molecular genetics and biophysical methods that paved the way for the detailed mechanistic understanding of bR function. Remarkably, shortly before the combination of FTIR (Bagley et al. 1982; Mantele et al. 1982; Rothschild and Marrero 1982; Rothschild et al. 1981; Siebert et al. 1983) and resonance Raman (RR) spectroscopy (Braiman and Mathies 1980; Smith et al. 1985) with the molecular biology of bR, advanced normal mode analyses and ab initio calculations had evolved (Tavan and Schulte 1986; Tavan et al. 1985). Thus, vibrational and electronic properties of the retinal chromophore Schiff base could be interpreted and their implications for the proton affinity of the retinal Schiff base derived. Such calculations required sufficient experimental information at atomistic detail to be of any value and strongly relied on the distinction between chromophore (seen mostly with RR spectroscopy) and protein vibrational features (seen additionally in FTIR spectroscopy). The complementarity of these approaches enabled specific band assignments and thus the further elucidation of the coupling between retinal structure on the one hand and protein conformation on the other (Braiman and Mathies 1982; Mantele et al. 1981; Rothschild and Marrero 1982; Rothschild et al. 1981; Siebert et al. 1982).

On this newly developing ground, the advent of site-specific mutagenesis of bR introduced by the Khorana group accelerated interdisciplinary synergies between research groups performing retinal chemistry, quantum chemistry, and spectroscopy to read out and interpret the functional consequences of chemical (mostly targeting retinal, in few cases also individual amino acids) and genetic modifications of bacterio-opsin or a combination of both. In these collaborations, the molecular biology of bR provided both a methodological linkage and a touchstone not only for the validation of the deduced mechanistic models of proton transport, but also for the advancement of molecular dynamics (MD) simulations and novel quantum chemical concepts as such (Grossjean et al. 1990; Nonella et al. 1991; Yadav and Poirier 1991; Zhou et al. 1993). In retrospect, the early work on both bR and the bovine photoreceptor rhodopsin, which nucleated around the Khorana group in the late 1980s and early 1990s, provided a template for many similar interdisciplinary research activities in vibrational analyses of other and eventually more complex membrane proteins. The enormous strength of the combination of vibrational spectroscopies with site-directed mutagenesis is best appreciated by the wealth of mechanistic knowledge on bacterial proton transport that was accumulated during the time span from the seminal work with the direct involvement of the Khorana lab starting in 1987 to the first electron diffraction-based structural model (Grigorieff et al. 1996), followed by the recording of X-ray diffraction from bR crystals in lipidic cubic phase (Landau and Rosenbusch 1996) and eventually the first X-ray structure (Luecke et al. 1999).

The Khorana group was directly involved in 29 vibrational spectroscopy studies of bR mutants mostly addressing the fate of the proton at the Schiff base linkage to lysine 216 using RR spectroscopy (Rothschild and Marrero 1982) and the role of carboxylates as potential counterions to the protonated Schiff base and as hydrogen-bonding partners using FTIR (Rath et al. 1993b). However, the first functional study of expressed, solubilized, and lipid-reconstituted bR mutants concerned the suspected role of internal tyrosine residues as elements of a then postulated tyrosine-based proton conduction wire. Although the proton translocation does not depend on any single of the total of 11 tyrosines (Mogi et al. 1987), tyrosine 185 was identified to become distinctly perturbed during the early steps of the bR photocycle (Braiman et al. 1988a, b). Despite relatively minor direct importance in elucidating the mechanism of proton transport, these experiments demonstrate convincingly the complementarity of mutagenesis and spectroscopic approaches, which is typical of many studies that grew around the Khorana group. For example, to address the mechanistic role of tyrosines, the first site-directed isotope labeling of a membrane protein was performed using the *E. coli* tyrosine amber suppressor tRNA amino-acylated with ring-deuterated tyrosine for in vitro expression of bR. FTIR studies of corresponding bR mutants with a “tyrosine—> ring-^2^H_4_-deuterated tyrosine” replacement obtained by in vitro expression and functional reconstitution enabled for the first time a site-specific vibrational assignment under full preservation of the protein structure and confirmed the unique structural perturbation of tyrosine 185 in early intermediates of the bR photocycle (Sonar et al. 1994).

Given the lack of evidence for a multi-tyrosine proton conductance in bR, carboxylates were quickly recognized as the prime candidates for transient protonation sites and were studied intensely by FTIR difference spectroscopy of bR mutants in which aspartic acid and glutamic acid residues were replaced by their amidated counterparts (Braiman et al. 1988a, b). The identification of the transfer of the Schiff base proton to its counterion aspartate 85 (Rath et al. 1993a; Rothschild et al. 1993) in the M intermediate of bR was a milestone in the mechanistic understanding of proton translocation. In a similar manner, the reprotonation of the Schiff base from the intracellular side during the M to N transition was identified with the help of FTIR difference spectroscopy in the K. J. Rothschild group using bR mutants from the Khorana lab (Bousche et al. 1991; Rothschild et al. 1993). FTIR difference spectroscopy was also used to measure the orientation of vibrational transition moments of retinal (Fahmy et al. 1991) as well as the presence of an extended water cluster interacting with specific amino acids at the proton release side of bR (Garczarek and Gerwert 2006). In addition, polarized FTIR spectroscopy was employed to the orientation of α-helical secondary structure relative to the plane of the purple membrane (Rothschild and Clark 1979).

Key bR mutants were also studied with advanced time-resolved spectroscopy methods to measure the kinetics of specific proton uptake and release reactions. For example, time-resolved photovoltage measurements of bR reconstituted into lipid vesicles adsorbed to black lipid membranes or bR-proteoliposomes attached to the surface of a phospholipid-impregnated collodion film were carried out using a 100-kHz differential AC conductivity apparatus to measure quantum yield and transient ion movements (Marinetti et al. 1989). Time-resolved laser flash photolysis spectrometry in conjunction with pH-sensitive fluorescent indicators showed that mutation of aspartic acid 96 to asparagine slowed the kinetics of M intermediate decay and the associated proton movement, suggesting that aspartic acid 96 was the internal proton donor for Schiff base reprotonation (Holz et al. 1989; Otto et al. 1989).

Since artificially reconstituted bR mutants were all in monomeric form, not in the trimeric crystalline form characteristic of bR in purple membrane, a method was developed to transform *H. halobium* with mutant genes that would result in the expression of trimeric bR mutants. This system was validated using X-ray diffraction of a cysteine-containing bR mutant and its mercury derivative to localize a specific amino acid residue in the extracellular loop of bR and thereby define the precise orientation of the monomeric units with the bR trimer (Krebs et al. 1993). Although this line of research was not pursued intensively, the interdisciplinary methodological development needed to carry out the work was emblematic of the forward-thinking strategy of the Khorana laboratory still after nearly 20 years of work on bR.

**Fig. 3:**
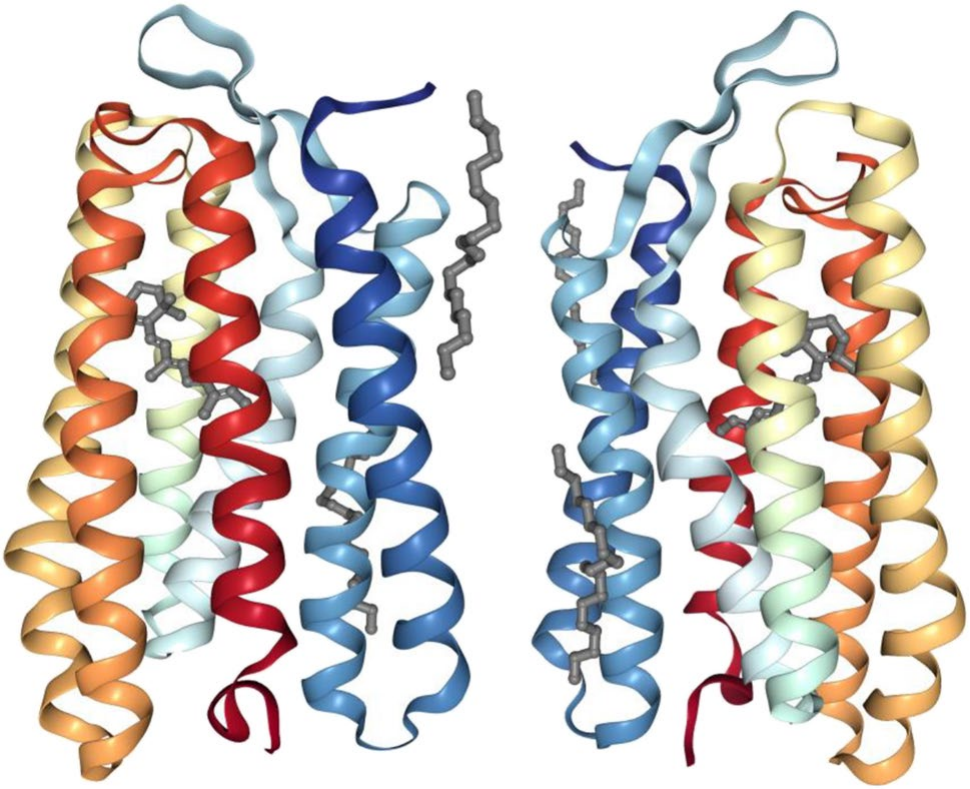
Representative structure of bR. Crystal structure Pdb:1KME (Faham and Bowie 2002) is shown as a ribbon illustration. The extracellular amino-terminal tail is toward the top, and the intracellular carboxyl-terminal tail is toward the bottom of the figure. The ribbon is rainbow colored from blue (amino-terminal tail) to red (carboxyl-terminal tail). The retinal chromophore is colored gray. The panel at the left shows a view from within the membrane bilayer focused on the transmembrane 1/8 interface, while the panel at the right shows a view rotated horizontally by about 180 degrees

The application of electron paramagnetic resonance (EPR) spectroscopy to the study of membrane proteins was pioneered in a major collaborative campaign between Khorana’s laboratory and the laboratory of W. L. Hubbell. Using EPR methods, it was possible to address local structural mobility (Steinhoff and Hubbell 1996) and eventually also distance changes between spin labels placed on different transmembrane segments. The basic strategy employed was to introduce individual cysteine residues into bR using site-directed mutagenesis. Since native bR does not contain cysteine residues, the substituted cysteine residues serve as useful chemical handles for the site-specific attachment of sulfhydryl-reactive nitroxide spin labels. In one notable example of the method, in a putative transmembrane segment of bR, cysteine residues were introduced at 18 consecutive positions, which were then labeled. EPR spectroscopy of the spin-labeled mutants was used to correlate freely diffusible oxygen quenching in the presence or absence of membrane-impermeant chromium oxalate. The periodicity of the oxygen accessibility for the consecutive cysteines was 3.6, suggesting a near perfect α-helical structure of the transmembrane segment. The data also allowed the formulation of a model in which the transmembrane segment could be properly oriented with respect to the rest of the protein (Altenbach et al. 1990), which was confirmed in subsequent high-resolution X-ray crystal structures (Fig. 3).

Additional EPR studies on engineered bR mutants tagged with spin labels reported movements in the second and third cytoplasmic loops coincident with the M to N transition where the proton donor side chain of aspartic acid 96 reprotonates the Schiff base (Steinhoff et al. 1994). Eventually, these movements in the loops were shown to be related to an outward movement of transmembrane helix F away from helices C and E (Rink et al. 2000). This relative helical displacement in the M to N transition assures both the change of accessibility of the Schiff base initially connected to the extracellular side of bR in the M1 state to its connectivity with the cytosolic (proton uptake) side in the M2 state and the concomitant positioning of the Schiff base for reprotonation in the N state.

The accumulation of mutagenesis-based knowledge on bR function influenced an entire generation of researchers, and much of the success in mechanistic understanding originated in the multitude of spectroscopic investigations of bR performed independently of the Khorana lab with different expression systems. The European branch of such studies nucleated in the Oesterhelt group, who was an early associate of Stoeckenius (Oesterhelt and Stoeckenius 1971, 1973), leading to extensive bR mutagenesis work starting in the late 1980s with the phenotype-based selection and sequencing of chemically induced *Halobacterium* species mutant strains (Soppa and Oesterhelt 1989). Early bR mutagenesis studies from the Oesterhelt group also included the use of vibrational spectroscopy (Fahmy et al. 1992). The studies on purple membrane and bR from several European groups, although not the focus of this review, extended to include a large variety of bacterial and algal retinal chromophore proteins and eventually led to the then unforeseeable application of the use of rationally designed channel rhodopsins, which are used today in optogenetics studies as recognized by the Lasker Award in 2021 (Rost et al. 2017).

## Studies of visual pigment rhodopsin

**Fig. 4:**
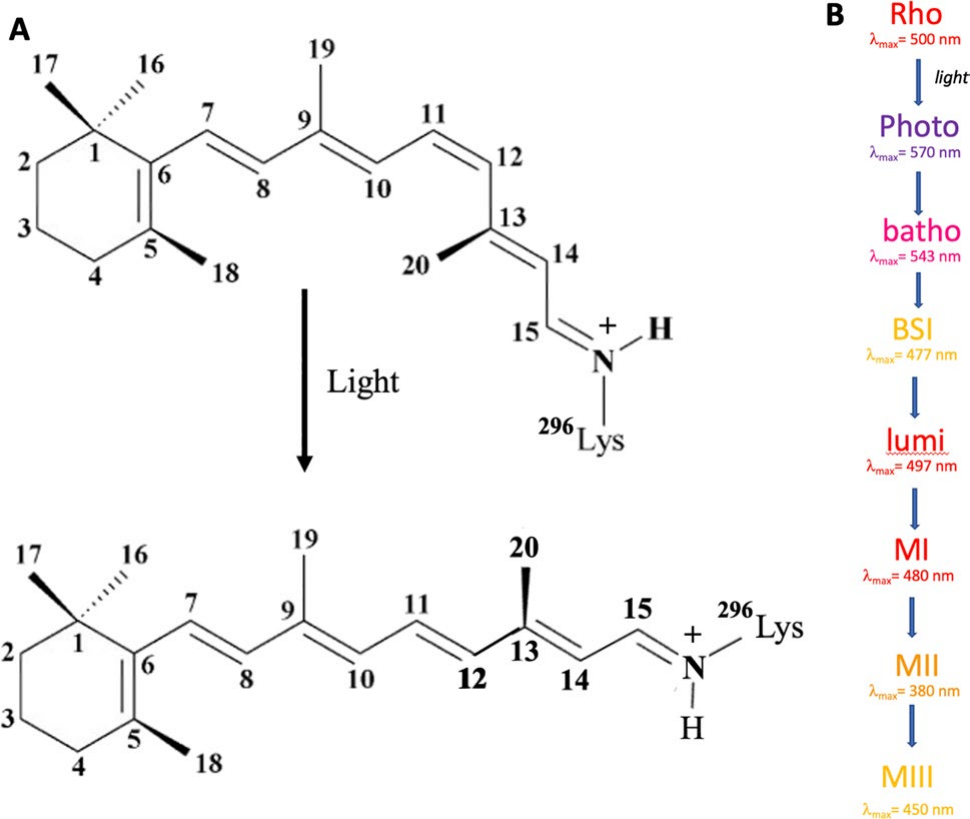
Principles of rhodopsin function. **A** The chromophore in rhodopsin is 11-*cis*-retinal, which photoisomerizes to all-*trans*-retinal. **B** The key intermediates in the photoactivation of rhodopsin are presented along with their wavelengths of maximal absorption. Abbreviations: photo, photorhodopsin; batho, bathorhodopsin; bsi, blue-shifted intermediate; lumi, lumirhodopsin; MI, metarhodopsin I; MII, metarhodopsin II; MIII, metarhodopsin III

As work on bR continued to elucidate the mechanism of light-driven proton pumping and how it coupled to the bR photocycle, a few members of Khorana’s group in the early to mid-1980s started work on another intrinsic membrane protein with a retinylidene chromophore, the visual pigment rhodopsin. Rhodopsin resides at high concentration in the disc membrane of the outer segment of the rod cell and is responsible for photon capture in dim-light vision. In the primary event in visual phototransduction, the 11-*cis*-retinylidene chromophore of rhodopsin absorbs a photon and photoisomerizes to all-*trans*-retinal (ATR) (Fig. 4). The photochemistry of visual pigments contrasts with that of bR where the dark-adapted ATR photoisomerizes to the 13-*cis*-retinylidene chromophore in response to light absorption. Photoactivated rhodopsin undergoes a series of thermal conformation changes resulting in the formation of metarhodopsin II (MII), which can engage a membrane-associated heterotrimeric guanine-nucleotide binding regulatory protein (G protein) called transducin, which releases bound GDP. The α subunit of transducin then takes up GTP and is released from rhodopsin to engage the γ subunit of a cGMP phosphodiesterase. This engagement causes the phosphodiesterase to become disinhibited, and cellular cGMP levels drop, which causes the closing of plasma membrane cGMP-gated cation channels. The resulting change in membrane cation conductance causes a relative hyperpolarization of the rod cell and a change in its rate of synaptic firing (Menon et al. 2001).

Rhodopsin is a prototypical member of a superfamily of cell surface receptors in animals called G protein–coupled receptors (GPCRs), which all share a seven-transmembrane helical topology in the membrane and couple to heterotrimeric G proteins to regulate cellular signaling pathways. GPCRs have evolved to bind a wide range of endogenous ligands, including biogenic amines, neurotransmitters, lipids, fatty acids, neuromodulatory peptides, proteins, odorants and certain tastants. GPCRs are also an important class of pharmaceutical drug targets in the human proteome, and about one-third of approved therapeutic agents engage GPCR targets. Recent advances in the study of structural biology of GPCRs, including reports of high-resolution X-ray crystal structures and cryo-electron (cryo-EM) single-particle structures of approximately 80 distinct GPCRs in complex with either inhibitory inverse agonists, antagonists, or agonists, have transformed the field. However, many early insights concerning the molecular mechanisms of GPCR-mediated transmembrane signal transduction came from interdisciplinary studies of rhodopsin that began with the development of methods to prepare relatively abundant and pure samples of rhodopsin from bovine retinas, in parallel with electrophysiological studies, many on isolated amphibian photoreceptors (Sakmar et al. 2002).The first high-resolution crystal structure of bovine Rho was not solved until 2000 (Palczewski et al. 2000), while the first crystal structure of a GPCR for a ligand-activated receptor, the β_2_-adrenergic receptor, was not reported until 2007 (Cherezov et al. 2007).

The beginning of the molecular biological studies of GPCRs began with the cloning of complementary DNA (cDNA) for bovine rhodopsin, which confirmed contemporaneous reports of its primary structure from amino acid sequencing (Hargrave et al. 1983; Nathans and Hogness 1983). Khorana’s team then designed and synthesized a full-length gene for bovine rhodopsin using automated oligonucleotide synthesis. Their synthesis strategy was to prepare a series of oligonucleotides on a solid support resin that were annealed batchwise to create overlapping double-stranded segments, which were then purified and ligated together to build up the full-length gene in segments (Ferretti et al. 1986). The synthesis of a gene for the α subunit of transducin was reported 2 years later (Sakmar and Khorana 1988). The synthetic rhodopsin gene was designed to facilitate site-directed mutagenesis using synthetic restriction fragment replacement, which was pioneered earlier in studies of bR. A key breakthrough was expression of the synthetic gene in mammalian COS-1 cells in tissue culture using a novel expression plasmid (Oprian et al. 1987). Mammalian GPCRs, including rhodopsin, are generally not able to be expressed in *E. coli* or purified in denatured form and refolded into functionally active receptors as was the case for bR. Expression of rhodopsin in mammalian cells was detected by immunoblot analysis using an anti-rhodopsin monoclonal antibody (mAb) called 1D4. Importantly, the immunopurification of the expressed recombinant rhodopsin pigment in detergent solution in functional form was accomplished using a 1D4 mAb immunoaffinity resin (Oprian et al. 1987). Over time, expression vectors and cell lines were improved, but the general method pioneered by D. D. Oprian in Khorana’s laboratory is still used extensively today to prepare myriad visual pigment and GPCR samples for biochemical, biophysical, and structural studies and is a key enabling methodology in the field.

Similar to the case with bR, FTIR vibrational spectroscopy had been well-established for the native bovine rhodopsin from natural sources (Bagley et al. 1985; Degrip et al. 1985; Ganter et al. 1988; Mantele et al. 1982; Siebert et al. 1983) as had RR spectroscopy (Deng and Callender 1987; Doukas et al. 1978; Eyring and Mathies 1979; Palings et al. 1987; Palings et al. 1989). The advent of the ability to make site-directed mutants of rhodopsin quickly allowed analogous questions of internal protein electrostatics provided by dipolar interactions of retinal with neighboring amino acids and by internal carboxyl groups to be addressed. RR spectroscopy was initially more challenging than with bR because of the low abundance of engineered bovine rhodopsins that was available from a mammalian expression system. A successful solution was provided by microprobe RR spectroscopy. Its first application to rhodopsin mutants (Lin et al. 1992) revealed the more extended interaction of the Schiff base counterion glutamic acid 113 (Sakmar et al. 1989; Zhukovsky and Oprian 1989) with retinal because vibrations of the C_12_ region of retinal were also affected by counterion replacements. The full complexity of the delocalized electrostatics between the retinal Schiff base and neighboring side chains, however, became fully appreciated only in 2003 when the unexpected switch of the counterion from glutamic acid 113 to glutamic acid 181 in the transition to the MI state was discovered by RR spectroscopy (Yan et al. 2003). More subtle local dipolar protein retinal interactions were shown by the same method to provide the molecular mechanism of spectral tuning based on multiple amino acid replacements that shifted the color sensitivity of bovine rhodopsin from green to blue by substituting amino acids from the human blue cone pigment (Kochendoerfer et al. 1999; Lin et al. 1998).

Whereas retinal properties were naturally at the focus of RR studies, conformational changes of opsin were studied in analogy to bR mostly with FTIR difference spectroscopy. Using aspartic acid to asparagine and glutamic acid to glutamine single- and double-replacement mutants of bovine rhodopsin, FTIR difference spectroscopy revealed the stable protonation of internal carboxyl groups of glutamic acid 122 and aspartic acid 83 in dark state rhodopsin and the active receptor conformation MII (Fahmy et al. 1993). On the other hand, a previously well-characterized carboxyl protonation band was eventually assigned to glutamic acid 113, revealing the proton transfer from the Schiff base to its primary dark state counterion upon formation of the active MII conformation (Jager et al. 1994). Breakage of the internal salt bridge between a protonated retinal Schiff base and its counterion was thus demonstrated to be a crucial step in linking retinal isomerization to protein function in both the M state of bR and the MII state of rhodopsin. Furthermore, the assignments of protonation states of internal carboxylic acid side chains in the membrane-embedded core of the receptor arising from the FTIR difference spectroscopy studies allowed the firm assignment of “initial states” of these groups in computational MD studies of rhodopsin (Huber et al. 2004) and later other GPCRs.

The functional phenotypes of counterion mutants, originally engineered for spectroscopic analyses of internal protein electrostatics, became particularly interesting for more general mechanistic features of GPCR activation. For example, shifting the carboxyl counterion from its original position 113 to 117 (a position homologous to the “counterion” for bound amine ligands in adrenergic receptors) led to a pigment that activated the cognate G protein and transduced a signal in a light-dependent fashion irrespective of the protonation state of the Schiff base in the active state (Fahmy et al. 1994). This observation demonstrated that it is the neutral state at position 113, rather than a neutral Schiff base, that promotes receptor activation after retinal photoisomerization. Similar to the identification of activity-promoting electrostatic interactions between retinal and opsin in rhodopsin mutants, the steric coupling to the retinal methyl groups at position 9 and 13 was addressed using a combination of chemical polyene modification, site-directed mutagenesis, and vibrational spectroscopies (Yan et al. 2004). Interestingly, “structural defects” in retinal that led to reduced receptor activation could be partially compensated by “allosteric” mutations as demonstrated by the partial rescue of MII formation in the 13-des-methyl-retinal–containing pigment upon replacement of the class-conserved glutamic acid 134 by glutamine, whereas the structural defect in the 9-des-methyl-retinal pigment could not be rescued (Vogel et al. 2006). The ensemble of these studies demonstrated the important role of the 9-methyl group of retinal in driving protein conformational changes after photoisomerization, particularly by interacting with glycine 121 (Han et al. 1997). These and earlier results supported the notion of receptor activation as a collection of binary local structural “on” and “off” states (Fahmy et al. 1995; Shieh et al. 1997).

Motivated by the strength of combining vibrational analyses of membrane protein function with site-directed chemical modification as pioneered by Khorana for bR (Sonar et al. 1994), genetic code expansion technology was also developed to facilitate the expression in mammalian cell lines of rhodopsin containing non-canonical amino acids. Using amber codon suppression, Ye and colleagues, including U. L. Rajbhandary, developed a highly efficient orthogonal amino acyl-tRNA synthetase/tRNA pair that was used to introduce *p*-benzoyl-phenylalanine or *p*-acetyl-phenylalanine site-specifically into the GPCR C–C chemokine receptor 5 (CCR5) (Ye et al. 2008). The system was later adapted for studies of rhodopsin in which *p*-azido-phenylalanine could be introduced site-specifically in rhodopsin to introduce local polarity probes specifically for FTIR spectroscopy (Ye et al. 2009). The system was used to determine the precise onset of the crucial movements of transmembrane helices 3, 5, and 6 during photoactivation of rhodopsin, which could be traced back to the metarhodopsin I intermediate, the precursor of the active state MII conformation (Ye et al. 2010).

As vibrational spectroscopy studies of rhodopsin were being advanced beginning in the mid-1990s, several other biophysical methods previously applied to bR were also used to study rhodopsin, including flash photolysis with rapid time-resolved spectroscopy in combination with linear dichroism to measure the kinetics, spectral properties, and chromophore structural changes in very early rhodopsin photointermediates (Jager et al. 1997a, b, c). Flash photolysis in conjunction with pH-sensitive fluorescence probes was also used to study engineered rhodopsin mutants to confirm the identity of glutamic acid 134 as the site of light-dependent proton uptake in the MII active state of rhodopsin (Arnis et al. 1994).

## Rhodopsin disease phenotypes

In contrast to studies on bR, rhodopsin mutagenesis was also motivated by human disease phenotypes. Among these, mutations responsible for congenital night blindness and for autosomal dominant retinitis pigmentosa (ADRP) played important roles in the use of vibrational spectroscopy for the elucidation of molecular mechanism. In both cases, the concept of local activation switches is quite instructive. A mutation in the gene for rhodopsin corresponding to a substitution of glycine 90 by aspartic acid (G90D) is found in one form of congenital night blindness, and the corresponding amino acid replacement was introduced into rhodopsin for in vitro molecular studies. FTIR spectra of G90D rhodopsin mutants revealed the partial anticipation of active state properties with respect to the Schiff base electrostatic environment and the dark state protein conformation, which exhibited properties otherwise seen only in the photoactivated state (Fahmy et al. 1996; Zvyaga et al. 1996). As a consequence of this partial anticipation of active state structures, the thermal threshold for receptor activation was lowered, suggesting that thermal receptor activation events might be occurring in the dark, which agrees with physiological and biochemical experiments (Sieving et al. 1995; Tian et al. 2017a, b).

Among the retinitis ADRP mutants, the role of glutamic acid 134 has been intensely investigated since an inherited mutation that results in its substitution by glutamine is associated with the disease. FTIR difference spectroscopy revealed the protonation of glutamic acid 134 in the active receptor state when this local switch is further stabilized by direct interaction with transducin (Fahmy 1998; Fahmy et al. 2000). Interestingly, the charge state of this cytosolic residue regulates the lipid insertion of the side chain, thereby rendering the MII formation sensitive to pH and lipid-protein interactions (Madathil and Fahmy 2009; Sandoval et al. 2016). The location of this carboxyl group in the aspartic acid (or glutamic acid)-arginine-tyrosine (Asp-Arg-Tyr, DRY) sequence in transmembrane helix 3 of class A GPCRs indicates that protonation-sensitive lipid-protein interactions may be common to class A GPCRs. “Lipid responses” to receptor activation had actually been evidenced previously by FTIR studies in the form of light-dependent lipid carbonyl stretching frequencies (identified with ^13^C-labeled 1-palmitoyl-2-oleoyl-*sn*-glycero-3-phosphocholine), which became visible only upon abolishing overlapping carbonyl vibrations of aspartic acid 83 and glutamic acid 122 by mutation (Isele et al. 2000). Such studies emphasize the complexity of membrane protein structural transitions that can be studied by the combination of FTIR spectroscopy and mutagenesis and which are difficult if not impossible to address otherwise.

Our current understanding of the molecular activation mechanism of rhodopsin as a paradigm for class A GPCRs is based on an enormous number of biochemical and biophysical in vitro experiments. Vibrational spectroscopies have contributed many mechanistic details which are inaccessible by high-resolution structure determination but are essential for the use of such structures in MD simulations or protein structure–based ab initio calculations. These details concern mostly the internal electrostatics provided by salt bridges and hydrogen-bond networks and their involvement in specific steps along the sequence of molecular activation processes. The existence and strength of such dipolar interactions has been “added” to atomic structures to build up consistent models of proton translocation and GPCR function in the case of rhodopsin. By the end of the 1990s, mutant visual pigments were being probed routinely using Raman spectroscopy, FTIR spectroscopy, flash photolysis, and rapid scanning UV–visible spectroscopy to elucidate the physicochemical mechanism of spectral tuning and to develop the concepts of “functional microdomains” and conformational coupling of independent allosteric domains in receptor activation.

## High-level functional expression of rhodopsin

A foundational advance in mammalian expression of rhodopsin in stable cell lines was reported in 1996, which eventually led to the ability to study engineered receptors using EPR spectroscopy and NMR spectroscopy (Reeves et al. 1996). Soon thereafter, the system was adapted so that labeling of rhodopsin with stable isotopes such as ^15^ N-lysine and ^13^C-glycine was possible (Eilers et al. 1999). Ultimately, enough material was available to support a major collaborative effort with S. O. Smith to carry out detailed magic-angle spinning solid state NMR studies of isotopically labeled rhodopsin mutants regenerated with chemically labeled synthetic chromophores to create artificial pigments. Collaborative studies using solid state NMR techniques provided important information about how specific amino acid residues and microdomains in rhodopsin coupled chromophore isomerization to receptor activation (Ahuja et al. 2009a, b; Patel et al. 2005). These studies also began to use structural information from X-ray crystallographic studies to begin to identify the functions of important structural features conserved in class A GPCRs. The Khorana laboratory also independently pioneered solution NMR studies of expressed rhodopsin. These efforts, especially the use of ^19^F-NMR to show light-induced changes in the structural mobility of specific domains in rhodopsin, were spearheaded by (Klein-Seetharaman et al. 1999a, 2002, 2004; Loewen et al. 2001).

Another long-lasting and notable collaborative effort was also initiated with W. L. Hubbell to extend the EPR spectroscopy studies on bR to rhodopsin mutants that were chemically tagged with nitroxide spin labels. These collaborative studies, carried out during nearly 15 years beginning in the mid-1990s, were facilitated by a general methodology called site-directed spin labeling in which native reactive, but non-essential, cysteine residues were removed from rhodopsin and then systematically reintroduced into the receptor and then labeled with sulfhydryl-reactive spin label tags. EPR spectra were correlated with the photoactivation states of a large collection of mutant receptors. Over time, essentially, the entire cytoplasmic surface of rhodopsin was mapped in this way to provide a very accurate picture of the dynamics of receptor conformational changes that were concomitant with receptor activation (Altenbach et al. 1999; Klein-Seetharaman et al. 1999b; Yang et al. 1996). The next iteration of the site-directed spin labeling methodology was to introduce pairs of cysteine residues to create dual spin-labeled rhodopsins that could be studied by double electron–electron resonance (DEER) EPR spectroscopy (Altenbach et al. 2001, 2008).

**Fig. 5:**
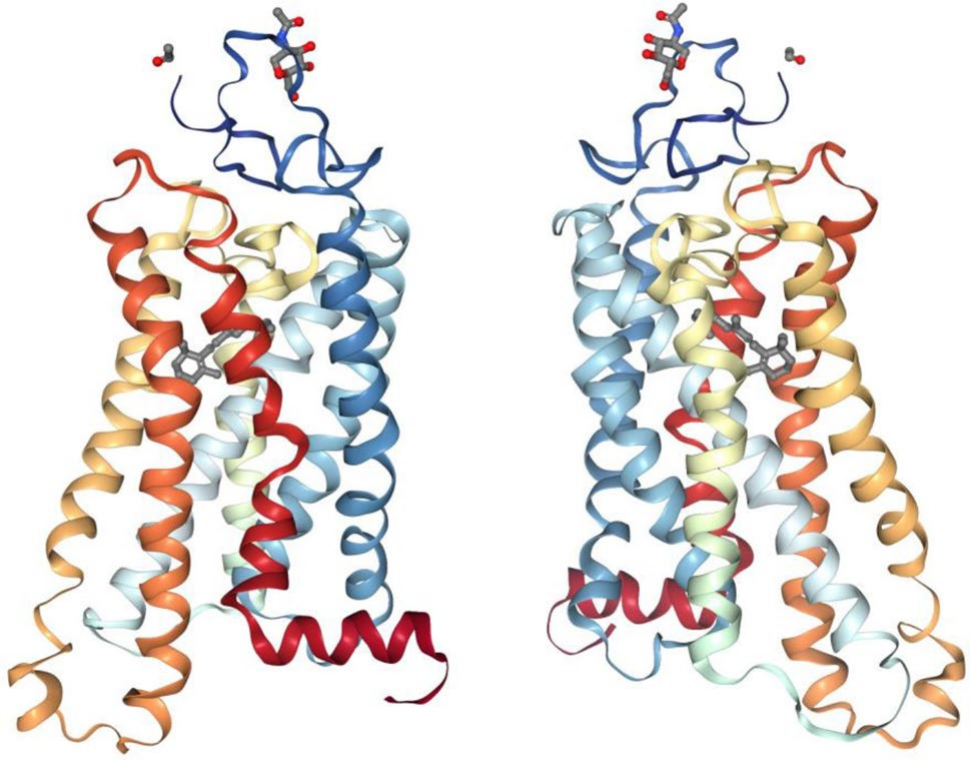
Representative structure of rhodopsin. Crystal structure Pdb:3C9M (Stenkamp 2008) is shown as a ribbon illustration. The extracellular amino-terminal tail is toward the top, and the intracellular carboxyl-terminal tail is toward the bottom of the figure. The ribbon is rainbow colored from blue (amino-terminal tail) to red (carboxyl-terminal tail). The retinal chromophore is colored gray. The panel at the left shows a view from within the membrane bilayer focused on the transmembrane 1/8 interface, while the panel at the right shows a view rotated horizontally by about 180 degrees

The most important discovery about rhodopsin activation from the spin label studies was probably the proposal that a rigid body movement of specific transmembrane segments was required for receptor activation (Farrens et al. 1996). Work in parallel carried out independently in other laboratories using site-directed mutagenesis, spectroscopy, and reversible chemical crosslinking approaches led to the helix movement model of GPCR activation that postulated that specific transmembrane helices, in particular transmembrane helix 6, moved away from the seven-helix bundle upon receptor activation (Sheikh et al. 1996). The change in the cytoplasmic surface conformation then facilitated proton uptake, leading to G protein binding and GDP nucleotide release. Proposed in 1996, the helix movement model was validated by X-ray crystal structures of opsin, MII, and then other ligand-activated GPCRs (Cherezov et al. 2007; Choe et al. 2011; Park et al. 2008; Scheerer et al. 2008) (Fig. 5).

## Concluding remarks

After having shared a Nobel Prize in 1968 for work on the genetic code that focused mainly on nucleotide chemistry and molecular biology, the decision of Khorana to commit fully to studies of membrane proteins in the early 1970s was bold and momentous. Over his entire career, he would end up spending more years of work focused on membrane proteins than he had on chemical biology of nucleotides. The decision to shift focus to membrane proteins came at an auspicious time when Khorana could apply molecular biology techniques to a new field to address the immense technical challenges that came with studying membrane proteins. Once heterologous expression systems were established for bR in *E. coli* and rhodopsin in mammalian cells in culture, methods were established for facile site-directed mutagenesis and purification of mutant proteins in functional form. The stage was then set for exhaustive structure–activity studies of both bR and rhodopsin. Khorana also committed major efforts to incorporate biophysical methods into the functional analysis of mutants in both systems. This commitment was transformational for two fields—understanding the mechanism of light-dependent proton pumping in bR and understanding the molecular mechanism of light-dependent transmembrane signaling in the visual pigment rhodopsin, which is a model system for the superfamily of GPCRs.

Khorana’s work on membrane proteins also included a clear vision of the functional implications of the membrane bilayer itself and the importance of lipid-protein interactions, as expressed, for example, in his biophysical papers from the early 1980s (Lind et al. 1981; London and Khorana 1982). Indeed, the functional role of lipid-protein interactions has become a research field in its own right with key concepts drawn from studies of retinal proteins (McKibbin et al. 2007). Lipid-protein interactions have now been studied in detail for their roles in the allosteric modulation of GPCR signaling (Baccouch et al. 2022; Jakubik & El-Fakahany 2021), and lipid scrambling activity has been shown for both retinal proteins and GPCRs (Khelashvili and Menon 2022; Menon et al. 2011; Verchere et al. 2017). In summary, the knowledge obtained from the innovative interdisciplinary biophysical studies on membrane proteins that Khorana and his colleagues and collaborators pioneered over nearly four decades was immense and foundational. Khorana’s influence on the entire field of studies of membrane proteins, and on the generation of scientists who worked with him, will be long-lasting.
